# Huge Atypical Meningioma in a 21-Month-Old Girl: A Case Report

**DOI:** 10.7759/cureus.71962

**Published:** 2024-10-20

**Authors:** Badr E Hafiz, Faisal Sukkar, Fares F Alsayegh, Mosab Abbas, Mohammed Aref

**Affiliations:** 1 Neurological Surgery, King Faisal Specialist Hospital and Research Centre, Jeddah, SAU; 2 Neurological Surgery, King Abdulaziz University Hospital, Jeddah, SAU; 3 Medicine, King Saud Bin Abdulaziz University for Health Sciences College of Medicine, Jeddah, SAU; 4 Neurosurgery, King Abdullah Medical Complex, Jeddah, SAU

**Keywords:** atypical, craniotomy, huge tumor, meningioma, pediatrics

## Abstract

Meningiomas are the most common intracranial tumors, but it's uncommon in the pediatric and adolescent populations. Pediatric meningiomas differ from adult meningiomas by a higher rate of malignant change, atypical location, male predominance and a higher recurrence rate. The most common presenting symptoms in supratentorial pediatric meningiomas are the signs and symptoms of high intracranial pressure (headache, vomiting, and nausea) and seizures. The most common presenting symptoms in infratentorial pediatric meningiomas are signs of cranial nerve deficits. Magnetic resonance imaging of the brain is the gold standard modality for diagnosis. The mainstay of management is surgical resection with the aim of gross total resection, as it is the strongest independent prognostic factor.

In this report, we describe an extremely rare case of gradual onset, progressive motor developmental delay in a 21-month-old girl with a huge right frontotemporal meningioma that was treated with surgical resection that, to the best of our knowledge, is the youngest in the literature.

## Introduction

Meningiomas are the most common intracranial tumors in adult patients. One-third of all primary central nervous system (CNS) tumors are caused by meningiomas, which make these tumors the most prevalent primary brain tumor [1]. The median age at diagnosis is usually 65 years, and it accounts for 36.5% of all diagnosed adult brain tumors and 1-5% of all diagnosed pediatric brain tumors [2]. Meningiomas have a gender preference, with females tending to have meningiomas three times more in comparison to males [3]. It originates from arachnoid cap cells or meningothelial dura tissue [3]. The gold standard grading in classifying histological and etiological meningioma factors is the World Health Organization (WHO) grading [4]. Meningiomas are categorized into 15 subtypes that are encompassed by three grades as per 2016 WHO guidelines: benign (grade I), atypical (grade II), and anaplastic (grade III) [4]. Forty-six percent of pediatric meningiomas are found in the cerebral convexities, 27% in the skull base, 10% intraventricular, and 7% account for pediatric spinal meningiomas [5]. Symptoms are location-dependent and vary from high intracranial pressure symptoms to specific functional area symptoms caused by mass effect, and given that supratentorial is by far the most common, they are usually present with seizures from cortical irritation [5]. Management can be divided into watchful waiting, surgical resection, and radiation therapy. The extent of surgical resection is the major clinical predictor of recurrence and overall survival in childhood meningiomas [6].

## Case presentation

**Figure 1:**
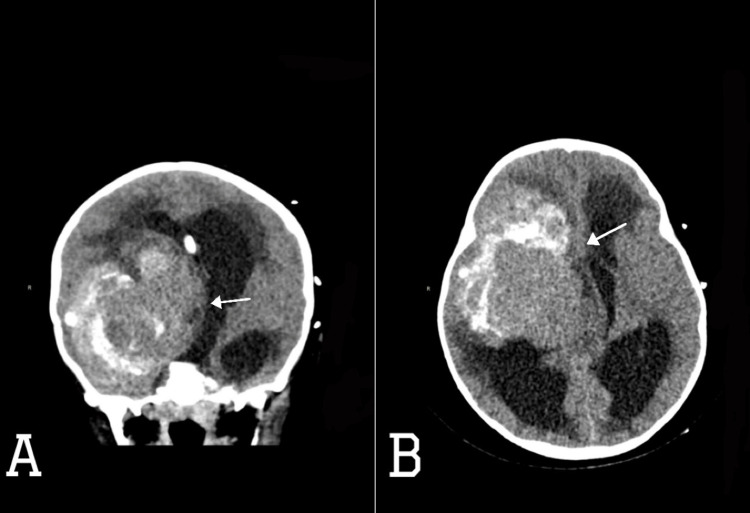
CT scan of the brain (A) axial and (B) coronal views showing right frontotemporal mixed iso-dense to hyper-dense mass measuring 6.7 x 7.5 x 7.1 cm in transverse x anteroposterior x craniocaudal diameters (white filled arrows), causing significant mass effect on and displacement of the right lateral ventricle frontal and temporal horns, third ventricle, right basal ganglia, right cerebral peduncle with 0.9 cm leftward midline shift, and active supratentorial hydrocephalus.

A 21-month-old twin girl who was a product of normal vaginal delivery was referred from a secondary hospital as a case of right frontotemporal brain lesion underwent left external ventricular drain (EVD). The patient's twin is also a girl, and she is healthy and keeping up with normal developmental milestones. She has six healthy siblings. Parents are consanguineous but not first or second-degree relatives. The patient has a history of regression in motor/speech developmental milestones. The parents also reported that the patient had multiple episodes of tonic seizures, and she had been sleeping and less active for one month. The patient was developmentally normal in comparison to her twin until one month ago when her parents noticed she became fatigued most of the day, unable to walk or talk, cried weakly, and vomited daily. An urgent computed tomography (CT) scan was done in an outside facility (Figure 1) and showed a right frontotemporal lesion causing midline shift, right lateral downward trans-tentorial herniation, and supratentorial obstructive hydrocephalus. 

**Figure 2:**
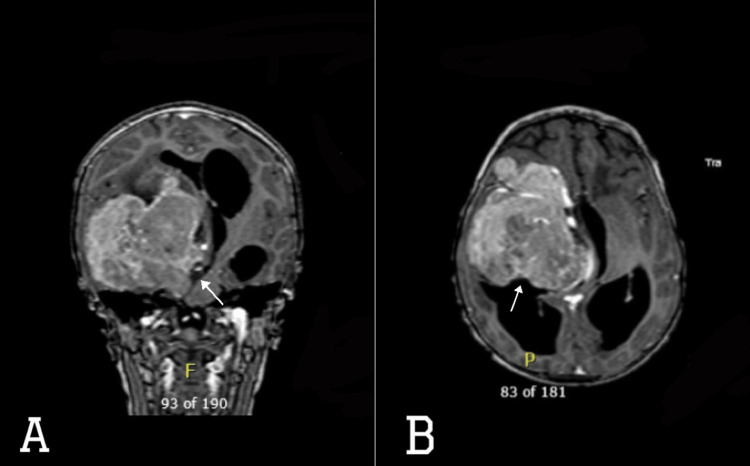
MRI brain with contrast (A) axial and (B) coronal views showing large lobulated right supratentorial frontotemporal mass with heterogeneous enhancement (white-filled arrows) on post-contrast images. It measures 6.7 x 7.5 x 7.1 cm (TR x AP x CC), causing significant mass effect on and displacement of the right lateral ventricle frontal and temporal horns, third ventricle, right basal ganglia, right cerebral peduncle, midbrain, pons, deep cerebral veins, right carotid terminus, right MCA, and right PCA. There is severe supratentorial hydrocephalus with CSF permeation (transependymal edema), 0.9 cm leftward midline shift, and right arteral downward trans-tentorial herniation MCA - middle cerebral artery, PCA - posterior cerebral artery; CSF - cerebrospinal fluid

An EVD was inserted in an outside facility, after which she markedly improved her level of consciousness and weakness. Twenty-three days later, she was transferred to our hospital. The parents declared that her activity and vomiting markedly improved after the EVD insertion, but she still has multiple episodes of tonic seizures. Examination revealed left-sided weakness Medical Research Council (MRC) power grade 3/5 in the left upper and 4/5 in the left lower limb, with no facial asymmetry. Pupils were bilaterally equally reactive at 3 mm. The patient appeared lethargic but otherwise was vitally stable. The patient was admitted and planned for surgical resection of the brain lesion. Magnetic resonance imaging (MRI) of the brain (Figure 2) was done and showed right frontotemporal heterogeneously enhancing mass, causing mass effect with midline shift and supratentorial hydrocephalus 

The patient underwent a right pterional craniotomy with tumor resection. The patient was positioned supine with the head rotated to the left. Right pterional craniotomy was performed, and dissection was advanced trans-sylvian. After debulking and careful detachment, the anterior cerebral artery (ACA), Middle cerebral artery (MCA), and optic nerve were identified and preserved. A segment of the lesion was found adherent to the right MCA and, therefore, minimal residual was left to prevent neurovascular injury. Subtotal (Simpson grade IV) resection of the tumor was achieved uneventfully without any intraoperative complications. The resected lesion was sent for histopathological analysis, which revealed foci of necrosis and increased mitotic activity (8/10 high power field (HPF)) with no brain invasion seen. The features suggestive of atypical meningioma which is classified as grade 2 by the recent 2021 World Health Organization (WHO) classification of central nervous system (CNS). 

**Figure 3:**
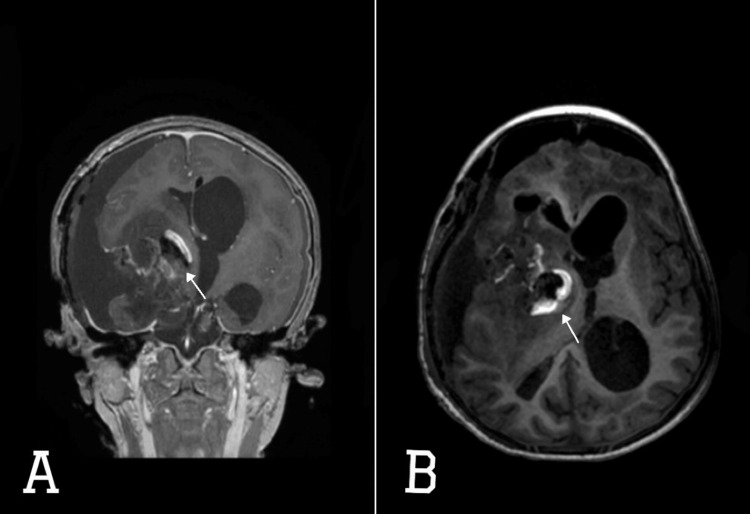
MRI brain with contrast (A) axial and (B) coronal views showing right frontoparietal craniotomy with tumor resection showing ending postoperative change with blood degradation product and enhancing rim (white filled arrows), edema, and air seen within surgical bed, right cerebral convexity fluid collection of maximal thickness of 1.3 cm, pneumocephalus in the right lateral frontal convexity and frontal horns, small layering of intraventricular hemorrhage seen at the right lateral occipital horns. No significant changes of the midline shift to the left side measures 1.1 cm. Slight improvement of the infratentorial uncal herniation. Improvement of the right lateral ventricle dilatation and mild decreased dilatation of the left lateral ventricle.

The patient was postoperatively shifted intubated to the pediatric intensive care unit (PICU), and her right eye was dilated 5 mm, and the left eye was 3 mm initially then both eyes became equal and reactive bilaterally 3mm. The patient was extubated on the second day postoperatively and shifted to the neurosurgical ward in a stable condition with the same weakness she had preoperatively on the left side. MRI of the brain postoperatively (Figure 3) was done and showed early postoperative changes with improvement in the right lateral ventricular dilatation and the infratentorial uncal herniation

**Figure 4:**
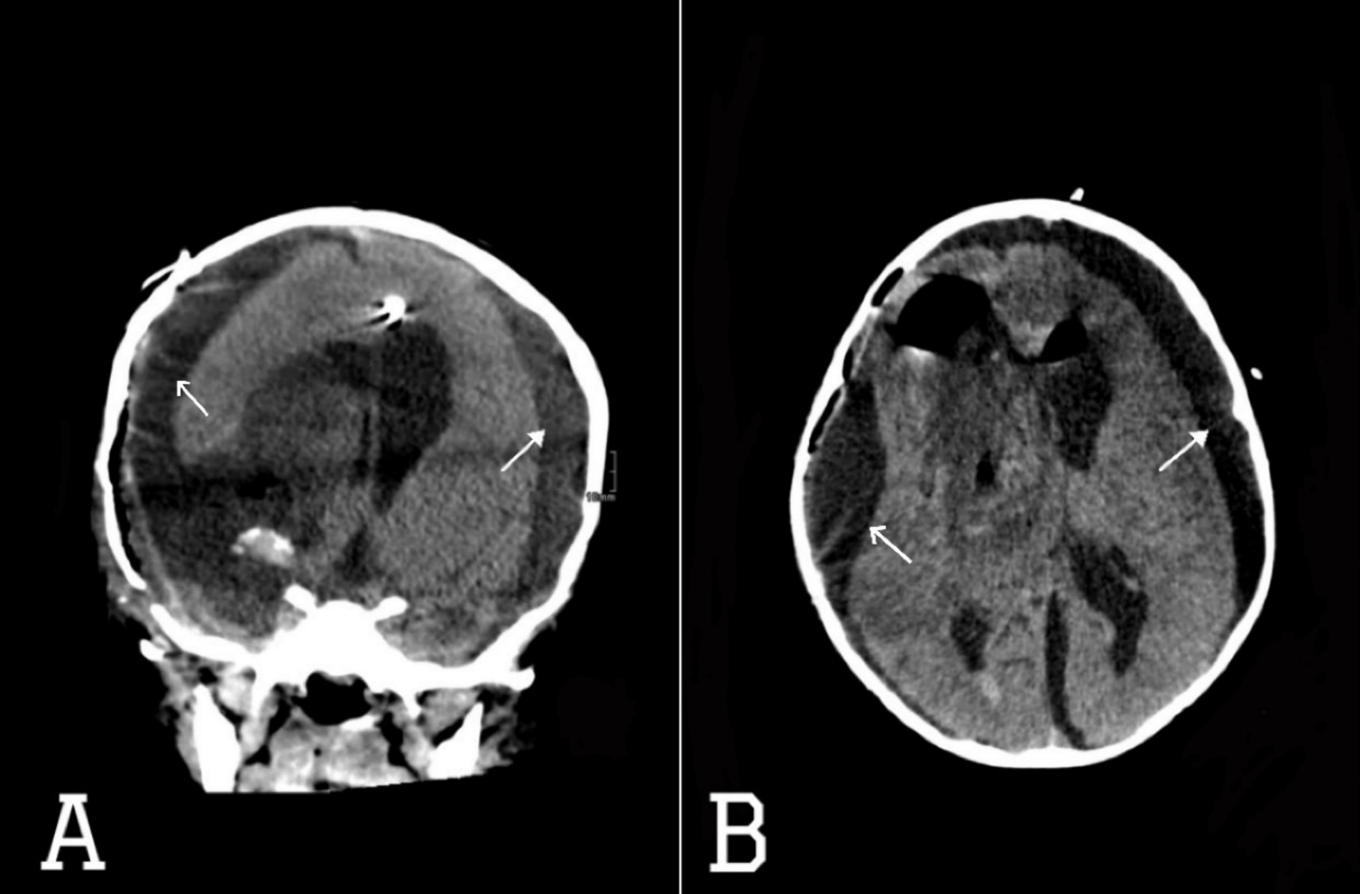
CT scan of the brain (A) axial and (B) coronal views showing postoperative changes of right frontoparietal craniotomy with newly developed left cerebral convexity subdural fluid collection of maximal thickness of 1.5 cm (white filled arrows) and interval increase in the right side subdural collection (white empty arrows), now measuring 1.8 cm compared to 1.3 cm previously. Interval decrease in size of the left lateral ventricle. Minor improvement of the left-sided midline shift and stable right uncal herniation.

On the sixth postoperative day, The patient developed weakness in the lower part of the right side of the face, which was purely an upper motor neuron lesion (UMNL). Two weeks later, the patient developed a fever, and a CT of the brain was done (Figure 4). It showed an increase in the right subdural fluid collection, a newly developed left subdural fluid collection for which a subdural drain was inserted, and an empirical antibiotic was started; however, cultures came negative.

**Figure 5:**
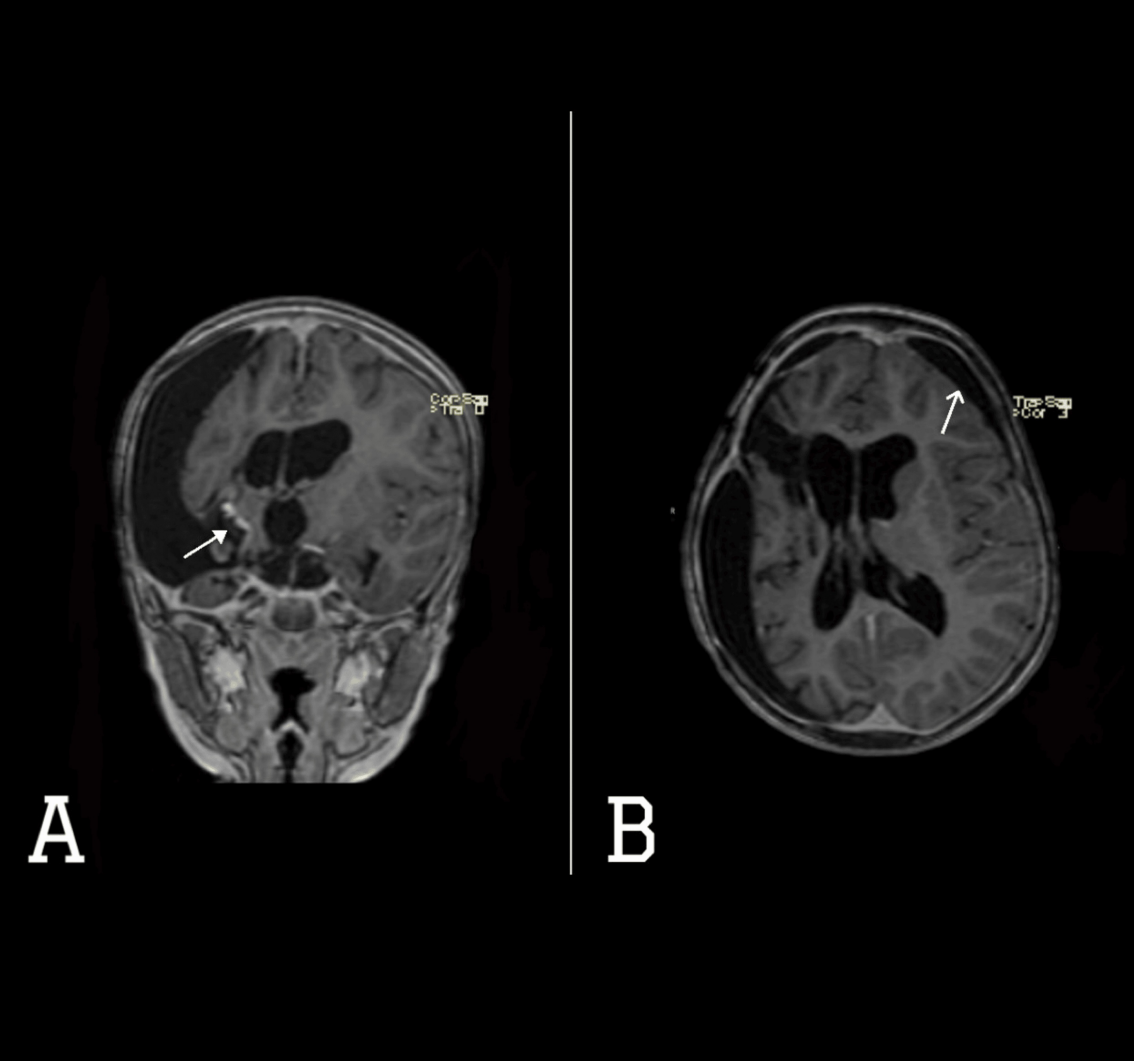
MRI brain with contrast (A) axial and (B) coronal views showing a redemonstration of right frontal parietal craniotomy for resection of the frontotemporal mass with a persistent enhancement of the dural reflections (white filled arrow) more on the right side. Encephalomalacia and gliosis involving the surgical cavity at the right frontal and temporal region marked a decrease in the left subdural fluid collection (White empty arrow) in comparison to the previous study. The prominent ventricular system showed a mild increase in comparison to the previous study likely related to a decrease in the mass effect related to the extra-axial collection.

The subdural drain was removed two weeks later after the fever subsided. The antibiotic course was continued for a total duration of four weeks. The patient's right facial UMNL is markedly improved, but it was the same as her baseline weakness in the left side, and she was discharged in a stable condition. Upon follow-up after 10 months in the clinic, the patient has no more episodes of vomiting, decreased activity, or seizures, and she has no active issues. Examination revealed that the right facial UMNL is totally recovered, the left upper limb power is improved and became MRC power grade 4/5, and the left lower limb is still the same 4/5. Her MRI brain follow-up (Figure 5) showed postoperative changes in the right frontotemporal craniotomy with a marked decrease in the subdural fluid collection in comparison to a previous study.

The patient underwent whole exome sequencing and its included screening for (BReast CAncer gene) BRCA1-associated protein-1 (BAP-1), multiple endocrine neoplasia-1 (MEN-1), neurofibromatosis type 2 (NF2), CREB-binding protein (CBP), leucine zipper-like post-translational regulator 1 (LZTR1) and SWIch/sucrose non-fermentable (SWI/SNF)-related matrix-associated actin-dependent regulator of chromatin subfamily B member SMARCB1 was negative. The patient will have continuous follow-up with serial imaging as an outpatient in the clinic to follow her progression and early detection of any new growth or recurrence as well as her clinical status.

## Discussion

Meningiomas are graded and classified by WHO into 15 subtypes in both pediatrics and adults [7]. Pediatric meningiomas can present as any of the 15 histopathological subtypes, as in adults, most classical morphologies (fibroblastic, transitional, and meningothelial) [7]. While grade I meningioma is the most common in both adults and pediatrics, a higher grade of meningioma occurs at a higher incidence in the pediatrics population in comparison to adults [7]. The most frequently reported genetic anomaly in pediatric meningiomas is monosomy of chromosome 22 [8]. Mutation of NF2 is the most recognized associated gene mutation with atypical meningiomas and is found in up to 75% of such tumors [8]. The diagnosis is usually based on imaging, starting with a CT scan of the brain, and then the imaging of choice is the MRI with contrast, which reveals the vivid enhancement [9]. Intrauterine detection is extremely rare, with some cases reported to be detected incidentally during routine fetal ultrasound as early as 20 weeks of gestation [9]. The definitive diagnosis of meningioma can be only confirmed by histopathological diagnosis postoperatively [9]. The management of meningiomas, as in any brain lesion, consists of three outlines: watchful waiting and observation with continuous follow-up, surgical reresection, and radiation [10]. The cornerstone and primary management strategy in pediatrics meningiomas is gross total resection, as it is considered the strongest independent prognostic factor [10,5]. Radiation therapy as an adjuvant treatment in pediatrics meningioma remains controversial [10]. The subsequent late toxic effect on the developing brain, along with the higher recurrence rate that necessitates surgical intervention in the pediatric age group, require case-based decision-making that is tailored to each patient considering the extent of resection, medical background, and current clinical status [10,8]. The long-term outcomes can be estimated by the location of the tumor, previous radiation exposure, extent of resection, and the presence of neurofibromatosis [10]. Meningioma in the pediatric age group has favorable outcomes in the majority [10].

To our knowledge, this is the youngest reported pediatric meningioma in the literature with such a huge size. There are only two more published case reports of pediatric meningioma in younger than five years patients in the literature [11]. 

## Conclusions

Pediatric meningiomas are slightly predominant in males and they are extremely uncommon below the age of three. They are rare in the first decade of life and extremely rare below the first three years of life. They are usually larger in size than the typical adult meningiomas and often with higher grades. The symptoms of pediatric meningiomas are non-specific and vary according to the location as well as the size causing the mass effect. The main factors attributed to recurrence are the extent of resection, histopathological grade, and the location of the lesion. Gross total resection is the cornerstone in management as it's the best prognostic factor and an important factor in the prevention of recurrence. Adjuvant radiotherapy in pediatrics is controversial, and it's case-based, considering the patient's medical background and current condition.
